# Enhanced Photoelectrochemical Activity of ZnO-Coated TiO_2_ Nanotubes and Its Dependence on ZnO Coating Thickness

**DOI:** 10.1186/s11671-016-1309-9

**Published:** 2016-02-24

**Authors:** Hua Cai, Peipei Liang, Zhigao Hu, Liqun Shi, Xu Yang, Jian Sun, Ning Xu, Jiada Wu

**Affiliations:** Department of Optical Science and Engineering, Fudan University, Shanghai, 200433 China; Key Laboratory of Polar Materials and Devices, Ministry of Education, East China Normal University, Shanghai, 200241 China; Institute of Modern Physics, Fudan University, Shanghai, 200433 China

**Keywords:** Photoelectrochemical activity, Heterogeneous nanostructure, TiO_2_, ZnO, ZnO-coated TiO_2_ nanotube

## Abstract

One-dimensional heterogeneous nanostructures in the form of ZnO-coated TiO_2_ nanotubes (ZnO/TiO_2_ NTs) were fabricated by atomic layer deposition of an ultrathin ZnO coating on electrochemical anodization-formed TiO_2_ nanotubes (NTs) with the thickness of ZnO coating being precisely controlled at atomic scale, and the photoelectrochemical activity of the fabricated ZnO/TiO_2_ NTs and the influence of ZnO coating and its thickness were studied. The structures of TiO_2_ NTs and ZnO coatings were characterized by X-ray diffraction, Raman backscattering spectroscopy, and transmission electron microscopy. The photoelectrochemical activity was studied through the measurements of electrochemical impendence, flat-band potential, and transient photocurrent density. The TiO_2_ NTs exhibit anatase structure, and the ZnO coatings are structured with hexagonal wurtzite. The photoelectrochemical activity of the ZnO/TiO_2_ NTs is strongly dependent on the thickness of ZnO coating. ZnO/TiO_2_ NTs with a thinner rather than a thicker ZnO coating exhibit better photoelectrochemical activity with reduced charge transfer resistance, increased negative flat-band potentials, and enhanced photocurrent densities. Under visible illumination, an increase of about 60 % in the photoelectrochemical activity is obtained for ZnO/TiO_2_ NTs with an about 2-nm-thick ZnO coating.

## Background

Titania (TiO_2_) and zinc oxide (ZnO) have recently attracted much attention because of their excellent optical, photoelectronic, and photoelectrochemical properties and consequently their potential applications in wide fields including photocatalytic reactions and photovoltaic processes [1–5]. Both metal oxides also have advantages of low cost, physical and chemical stability, nontoxicity, and ease of availability. In addition, TiO_2_ and ZnO have similar band-gap energies (~3.22 eV for TiO_2_ and ~3.37 eV for ZnO) and are compatible to each other to compose heterogeneous materials. A variety of heterostructured materials composed of TiO_2_ and ZnO (TiO_2_-ZnO) have been reported. Heterogeneous TiO_2_-ZnO materials exhibit improved individual and combined properties which are very distinct from those of the single constituting materials TiO_2_ and ZnO and are promising for applications such as photocatalytic reactions and photovoltaic processes. Dye-sensitized solar cells using ZnO-coated nanocrystalline TiO_2_ films as electrodes, for example, have shown enhanced conversion efficiencies [6]. In particular for heterostructured TiO_2_-ZnO, nanoscaled TiO_2_ and ZnO are superior to bulk and film materials in their large surface-to-volume ratio for a large interface between TiO_2_ and ZnO. Therefore, nanosized heterostructures constructed of nanoscaled TiO_2_ and ZnO show better photoelectronic, photochemical, and photoelectrochemical properties. When used as a photocatalyst or a photoelectrode, for example, heterogeneous ZnO-TiO_2_ nanostructures can provide improved performance due to the combination of the high activity of TiO_2_ with the high electron mobility of ZnO. Furthermore, the alignment of the staggered band gaps of TiO_2_ and ZnO is favorable for the separation of electrons and holes and consequently the suppression of electron-hole recombination [7, 8]. The staggered band alignment also results in an extension for spectral range of photoresponse, which allows an efficient use of light with a wide spectral band [9, 10]. Therefore, better electrochemical and photoelectrochemical properties and hence enhanced photocatalytic activities and increased photovoltaic efficiencies can be expected for the heterogeneous ZnO-TiO_2_ nanostructures because of their improved charge conductivity, enhanced charged carrier separation, and extended photoresponse range [11, 12]. Of the diverse ZnO-TiO_2_ nanoheterostructures, one-dimensional (1D) nanogeometry has received particular attention in the photocatalytic and photovoltaic applications due to its superior charge transport ability and short recombination pathway, which significantly suppresses the recombination of photogenerated electrons and holes [13–16]. The ultraviolet (UV) photoconductivity and photosensitivity of TiO_2_-coated ZnO nanorods have been reported to be largely enhanced [17]. Using ZnO-sheathed TiO_2_ as photoanodes, dye-sensitized solar cells have shown enhanced performance [18]. ZnO-coated TiO_2_ nanotube (ZnO/TiO_2_ NT) is one of the most studied 1D heterogeneous ZnO-TiO_2_ nanostructures [16, 19–21]. However, the optimized photocatalytic activity and photovoltaic efficiency of heterogeneous TiO_2_-ZnO materials including 1D heterogeneous ZnO-TiO_2_ nanostructures are strongly dependent on the morphology and geometry.

In comparison to bare TiO_2_ NTs, TiO_2_ NTs covered by a thin ZnO coating usually present improved electrochemical and photoelectrochemical properties and are expected to have high quantum efficiencies for photocatalytic reactions and photovoltaic processes [16, 19–21]. As a nanoscaled heterostructure, it has been proved that the properties of ZnO/TiO_2_ NTs are strongly dependent on the geometry and morphology of the heterogeneous nanostructure, the crystal structure of TiO_2_ and ZnO, and the interfacial quality between TiO_2_ and ZnO. In the present work, ZnO/TiO_2_ NTs were fabricated by atomic layer deposition (ALD) of a ZnO coating on the walls of electrochemically anodized TiO_2_ NTs, and the thickness of ZnO coating was precisely controlled at a sub-nanometer level by varying the growth cycle of ZnO during the ALD process. The properties of the fabricated ZnO/TiO_2_ NTs and their dependence on the ZnO coating thickness were studied for optimizing the electrochemical and photoelectrochemical activities.

## Methods

The method and the equipment used for sample fabrication have been described previously [22]. Through an electrochemical anodization process, vertically aligned TiO_2_ NTs were first formed on a polished Ti foil (99.99 % in purity, 0.1 mm in thickness). The formed TiO_2_ NTs were annealed at 450 °C in air for 3 h. A thin ZnO coating was then deposited on the walls of the TiO_2_ NTs by means of ALD using Zn(CH_2_CH_3_)_2_ (diethylzinc (DEZ)) and deionized H_2_O as zinc and oxygen precursors, respectively. The deposition of the ZnO coating was performed at 200 °C and the growth of the ZnO coating on the high-aspect-ratioed TiO_2_ NTs was governed by self-limiting reactions [23, 24]

1 $$ \mathrm{Zn}\mathrm{O}\mathrm{H}* + \mathrm{Z}\mathrm{n}{\left({\mathrm{CH}}_2{\mathrm{CH}}_3\right)}_2\to\ \mathrm{Z}\mathrm{n}\mathrm{O}-{\mathrm{ZnCH}}_2{\mathrm{CH}}_3* + {\mathrm{CH}}_3{\mathrm{CH}}_3, $$

2 $$ {\mathrm{ZnCH}}_2{\mathrm{CH}}_3* + {\mathrm{H}}_2\mathrm{O}\ \to\ \mathrm{ZnOH}* + {\mathrm{CH}}_3{\mathrm{CH}}_3 $$

The thickness of the deposited ZnO coating was precisely controlled by ALD growth cycles with 1 cycle of 0.5-s DEZ pulse, 2-s N_2_ purge, 0.5-s H_2_O pulse, and 2-s N_2_ purge. Several sample groups of ZnO/TiO_2_ NTs were prepared with the same TiO_2_ NTs but different ZnO coating thickness. Two of them, one with a 10-cycle deposit of ZnO coating (refereed as 10-cycle ZnO/TiO_2_ NTs) and the other with a 25-cycle deposit of ZnO coating (refereed as 25-cycle ZnO/TiO_2_ NTs), were compared in this work for a study of the influence of ZnO coating thickness on the properties including photoelectrochemical activity. The obtained heterogeneous nanostructured ZnO-coated TiO_2_ NTs samples were rinsed with deionized water and annealed at 450 °C for 30 min in air.

The sample morphology was examined by field-emission scanning electron microscopy (FESEM) with a Hitachi S-4800 microscope. The morphology and microstructure of the samples was characterized with a transmission electron microscope (TEM, Tecnai G^2^ F20 S-Twin). The composition and thickness of the ZnO coatings were evaluated by Rutherford backscattering (RBS) method using a 2.0-MeV He^+^ beam at 165 ° scattering geometry. The He^+^ beam generated by a 9SDH-2 tandem accelerator at Fudan University was collimated to a diameter of 1.0 mm for RBS measurements. The structure of the samples was characterized by X-ray diffraction (XRD) with a Rigaku D/max-γ B X-ray diffractometer using a Ni-filtered Cu Kα radiation. The sample structure was also characterized through the analysis of vibrational modes by means of Raman backscattering spectroscopy which was performed with a Jobin-Yvon LabRAM HR 800 UV micro-Raman spectrometer using a 325-nm He–Cd laser light to excite the samples. UV–visible absorption spectra of the samples were recorded with a UV–visible spectrophotometer (Shimadzu UV-350) using a diffuse reflectance integrating sphere.

Photoluminescence (PL) measurements were carried out at room temperature (300 K) and a reduced temperature of 20 K by exciting the samples with a nonpolarized 325-nm He–Cd laser beam, dispersing the emitted luminescence with a 0.5-m spectrometer (Spectra Pro 500i, Acton), and recording the PL spectra with an intensified charge-coupled device (ICCD, iStar DH720, Andor). The spot size of the 325-nm laser beam exciting the samples for PL measurement is about 1 mm in diameter. The electrochemical properties of the samples were studied by electrochemical impedance and flat-band potential measurements using a Potentionstat/Galvanostat (EG & G, 273 A) and a two-phase lock-in amplifier (EG & G, 5210). In a three-electrode cell with a 0.5 M Na_2_SO_4_ solution, the impedance measurements were carried out at 0.0 V versus the reference electrode Ag/AgCl with frequencies ranging from 100 kHz to 0.1 Hz, and the flat-band potentials of the samples were determined from the Mott–Schottky plots obtained by measuring at 100 Hz with potentials scanning from 1.0 to 1.5 V at 50 mV/s. Linear-sweep cyclic voltammetry was performed at a potential ranging from +1.5 to −1.5 V versus Ag/AgCl in 0.5 M Na_2_SO_4_ at a scan rate of 50 mV/s. With an active area of 1 cm^2^, the samples were used as photoelectrodes and subjected to intermittent irradiation of visible light (100 mW/cm^2^) for photocurrent (PC) density measurements in the three-electrode cell in 0.5 M Na_2_SO_4_ using a CHI electrochemical analyzer (CHI 660A). A 500-W xenon lamp was used as the light source to provide visible light by removing infrared light with a quartz wafer filter and cutting ultraviolet (UV) light using a high-pass filter with a cutoff of 380 or 420 nm.

## Results and Discussion

**Fig. 1:**
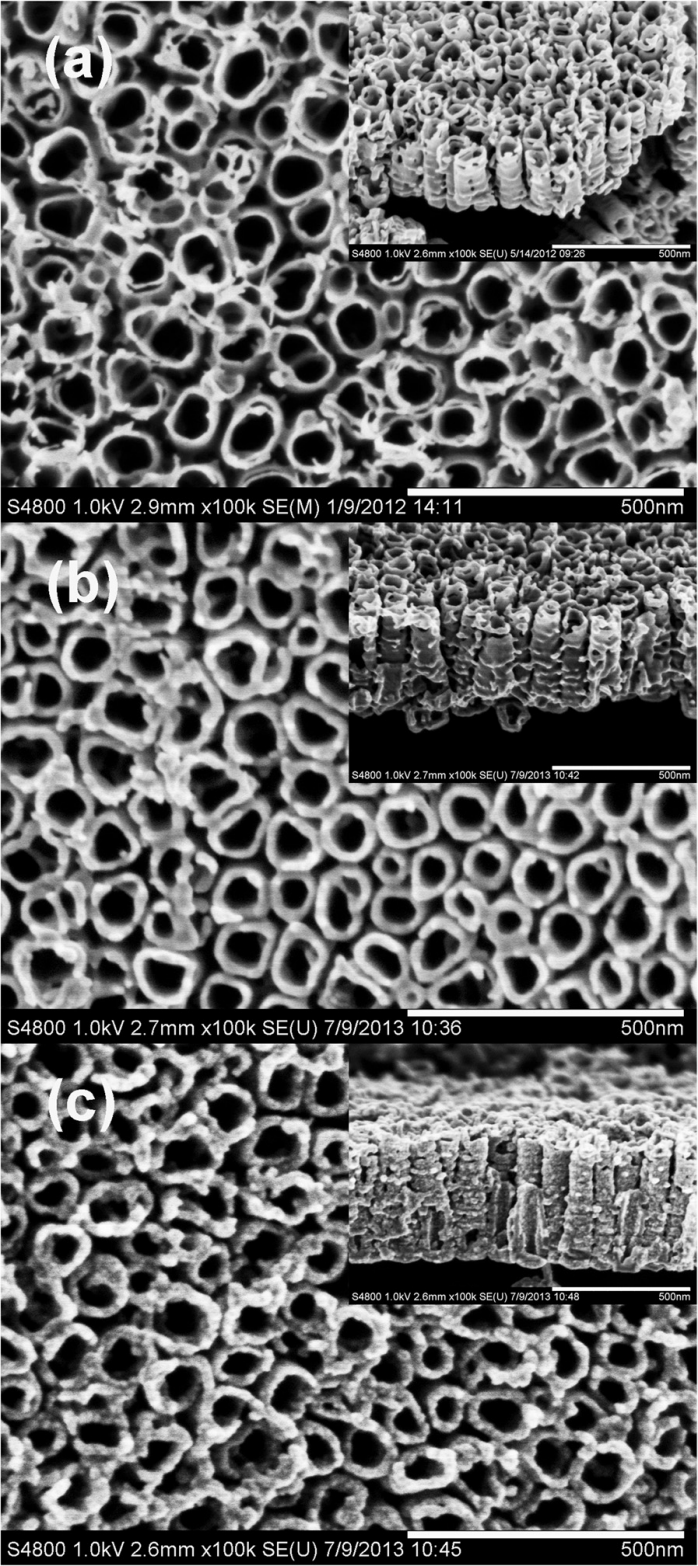
Top-view and cross-sectional SEM images of **a** bare TiO_2_ NTs, **b** 10-cycle ZnO/TiO_2_ NTs, and **c** 25-cycle ZnO/TiO_2_ NTs

Figure 1 shows typical planar and cross-sectional FESEM images of the samples. Figure 1a indicates that highly ordered and vertically aligned TiO_2_ NTs having an average diameter of ~60 nm and a wall thickness of ~15 nm were formed on Ti foil by electrochemical anodization process. Figure 1b, c shows the planar and cross-sectional FESEM images of ZnO-coated TiO_2_ NTs fabricated by depositing 10 or 25 cycles of ZnO on TiO_2_ NTs. It can be seen that the TiO_2_ NTs are fully and uniformly covered by the ZnO coatings on the top and the side walls, even deep down to the bottom of the tubes. The ZnO-coated TiO_2_ NTs have the same shapes as the bare TiO_2_ NTs, but with slightly reduced tube diameters and thickened tube walls. Generally, the 25-cycle ZnO-coated TiO_2_ NTs have smaller diameters and thicker walls than the 10-cycle ZnO-coated TiO_2_ NTs.

**Fig. 2:**
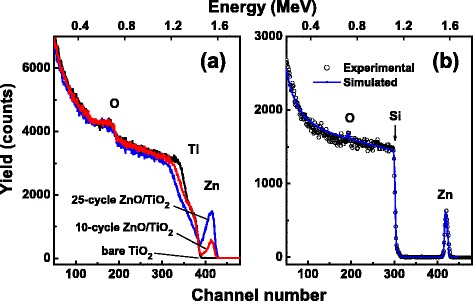
**a** RBS spectra of bare TiO_2_ NTs, 10-cycle ZnO/TiO_2_ NTs, and 25-cycle ZnO/TiO_2_ NTs. **b** Experimental (*circle*) and simulated (*solid line*) RBS spectra of a ZnO film grown on a Si substrate by ALD with 50-cycle deposit of ZnO

Figure 2a shows the random RBS spectra of the bare TiO_2_ NTs and the ZnO-coated TiO_2_ NTs. Due to the nanosized tubular structure of the samples, the signals from the scattered helium atoms have no clear and steep edges. The signals attributed to O atoms in the samples are weak, and the resolution is limited by the overlap of its peaks on the base spectra of the heavy element Ti in the underlying Ti foil. The presence of TiO_2_ NTs on Ti foil can be evidenced by the decreasing of the Ti signal near the Ti background edge. The salient peaks in the channel number ranging from 390 to 430 in the spectra of the ZnO-coated TiO_2_ NTs are related to the contribution from Zn atoms in the ZnO coatings covering the TiO_2_ NTs. The coverage of the TiO_2_ NTs by ZnO coatings also results in further decreasing of the Ti signal near the Ti background edge. In comparison to the spectrum of the 10-cycle ZnO/TiO_2_ NTs, the higher yield attributed to Zn in the spectrum of the 25-cycle ZnO/TiO_2_ NTs indicates the thicker ZnO coating of the 25-cycle ZnO/TiO_2_ NTs. However, the thickness and the composition of the ZnO coatings cannot be calculated from the spectra shown in Fig. 2a because of the complicated profiles of the spectra taken from the nanosized tubular-structured samples. RBS analysis was also carried out on a ZnO film grown on a polished Si (100) substrate by ALD with 50-cycle deposit of ZnO with the other conditions remaining the same as for depositing ZnO coatings on the TiO_2_ NTs. The RBS spectrum recorded for the ZnO film on Si is shown in Fig. 2b. The SIMNRA simulation [25] of this spectrum reveals that the ZnO film has a stoichiometric composition and a thickness of 10.25 nm, from which it is deduced that the thickness of 1-cycle deposit of ZnO is about 0.21 nm, consistent with the result obtained by spectroscopic ellipsometry performed for a ZnO thin film on a Si substrate deposited by the same method and conditions, which gave an estimated thickness of 0.225 nm for 1-cycle deposit of ZnO [26]. Therefore, the 10-cycle ZnO/TiO_2_ NTs and 25-cycle ZnO/TiO_2_ NTs can be estimated to have a ZnO coating of about 2.10 and 5.25 nm in thickness, respectively.

**Fig. 3:**
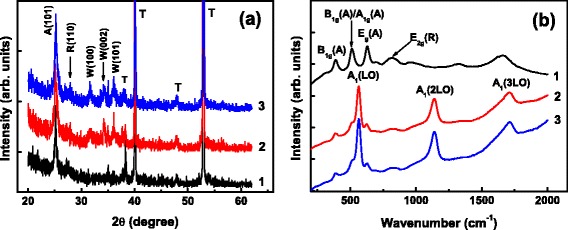
**a** XRD patterns and **b** Raman scattering spectra of bare TiO_2_ NTs (*1*), 10-cycle ZnO/TiO_2_ NTs (*2*), and 25-cycle (*3*) ZnO/TiO_2_ NTs. The XRD patterns and Raman scattering spectra are moved vertically for clarity

Figure 3a illustrates the XRD patterns of the bare TiO_2_ NTs and the TiO_2_ NTs covered by ZnO coatings. Besides the diffractions from the Ti foil (denoted by T), a prominent diffraction peak with 2*θ* at 25.22 is identified for the samples whether covered by ZnO or not. This peak is indexed to the (101) diffraction of anatase TiO_2_ (denoted by A in Fig. 3a) (JCPDS 21-1272). For the bare TiO_2_ NTs, in addition, a weak peak near 27.40 can be recognized, which could be assigned to the (110) diffraction of rutile TiO_2_ (denoted by R) (JCPDS 21-1276). The TiO_2_ NTs are therefore of nearly tetragonal anatase phase with minor rutile phase. For the TiO_2_ NTs covered by a 10-cycle deposit of ZnO, three additional peaks appear, which can be ascribed to hexagonal wurtzite ZnO (denoted by W) and are related to the diffractions from the (100), (002), and (101) orientations of wurtzite ZnO (JCPDS: 36-1451), respectively. The multidiffractions reveal the non-oriented growth of ZnO on the TiO_2_ NTs. For the TiO_2_ NTs covered by a 25-cycle deposit of ZnO coating, the diffractions ascribed to wurtzite ZnO increase with a reduced peak width, indicating an improvement in the crystallinity of the ZnO coating with the coating thickness increasing. The above XRD results reveal that fabricated ZnO/TiO_2_ NTs are constructed of tetragonal anatase TiO_2_ NTs and hexagonal wurtzite ZnO coatings.

Raman backscattering measurements confirm the anatase structure of the TiO_2_ NTs and the wurtzite structure of the ZnO coatings, as shown in Fig. 3b. The Raman spectrum taken from the bare TiO_2_ NTs exhibits three distinct peaks located at 392, 514, and 633 cm^−1^, respectively. They are attributed to the characteristic Raman reactive B_1g_ mode, doublet A_1g_/B_1g_ mode, and E_g_ mode of anatase TiO_2_ [27, 28]. In addition, a broad band centered near 820 cm^−1^ is resolved, which is attributed to the B_2g_ mode of rutile TiO_2_. In the Raman spectrum taken from the bare TiO_2_ NTs, A and R in parentheses denote the anatase and the rutile phase of TiO_2_, respectively. After being covered by a 10- or 25-cycle deposit of ZnO coating, the above TiO_2_ modes are greatly suppressed and the Raman spectra are predominated by three strong backscattering peaks at 574, 1147, and 1720 cm^−1^. The strongest peak at 574 cm^−1^ is attributed to the polar optical mode associated with longitudinal optical (LO) phonons of ZnO [A_1_(LO)], while the other two peaks at 1147 and 1720 cm^−1^ are the overtones of A_1_(LO) and result from 2- and 3-phonon scattering processes [A_1_(2LO) and A_1_(3LO)], respectively [29, 30]. The reduction in the measured Raman signals scattered from TiO_2_ for the ZnO-coated TiO_2_ nanotubes is mainly attributed to the absorption of the photons in the exciting 325-nm light by the ZnO coating.

**Fig. 4:**
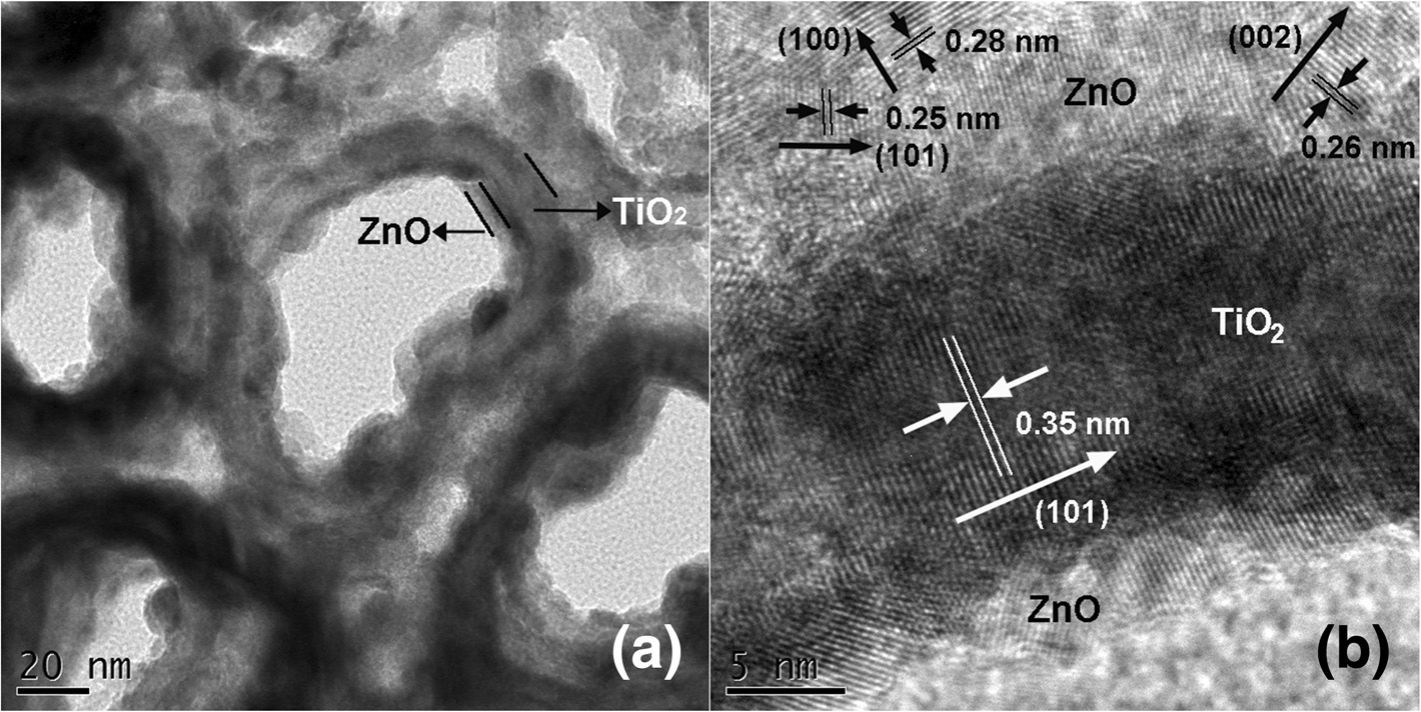
Representative **a** TEM and **b** high-resolution TEM images of 25-cycle ZnO/TiO_2_ NTs

The morphology of the ZnO/TiO_2_ NTs and the crystal structure of the TiO_2_ tubes and the ZnO coatings are further confirmed by TEM and high-resolution TEM examination, as shown in Fig. 4 which depicts representative TEM and high-resolution TEM images taken from the 25-cycle ZnO/TiO_2_ NTs. The heterogeneous structure can be seen clearly from the images shown in Fig. 4a, b. We have measured the wall thickness of TiO_2_ tube yielding ~15 nm which is consistent with the result obtained by FESEM, and the thickness of ZnO coating on the inner-wall of TiO_2_ tube giving ~5 nm, consistent with that estimated by RBS. The high-resolution TEM image evidences the crystal structure of the TiO_2_ tube and the ZnO coating. The interplanar spacings were determined for both TiO_2_ and ZnO. We can see from Fig. 4b that TiO_2_ is nearly single crystalline with interplanar spacing of 0.35 nm which is in agreement with *d*_101_ of anatase TiO_2_. Figure 4b also reveals several orientations of ZnO. Their interplanar spacings were determined to be 0.28, 0.26, and 0.25 nm, respectively, matching well with *d*_100_, *d*_002_, and *d*_101_ of wurtzite ZnO. The crystal structure of the sample characterized by high-resolution TEM is in conformity with the XRD characterization.

**Fig. 5:**
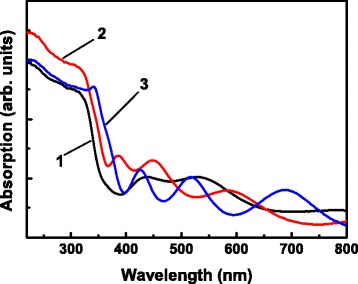
UV–visible absorption spectra of bare TiO_2_ NTs (*1*), 10-cycle ZnO/TiO_2_ NTs (*2*), and 25-cycle (*3*) ZnO/TiO_2_ NTs

The recorded UV–visible absorption spectra of the samples are displayed in Fig. 5. It can be seen that compared with the bare TiO_2_ NTs, the ZnO-coated TiO_2_ NTs exhibit an obvious red shift in the absorption edge, indicating that the ZnO coating on the TiO_2_ NTs extends the photoresponse to longer wavelength, and the red shift increases with the ZnO coating thickness increasing. The red shift in the absorption edge of heterostructured materials composed of TiO_2_ and ZnO has been reported and can be attributed to the staggered band gaps of TiO_2_ and ZnO and the interface effect between TiO_2_ and ZnO [9, 31, 32]. The red-shifted absorption edge and consequently the extended photoresponse region allow the ZnO-coated TiO_2_ NTs to work in the visible region.

**Fig. 6:**
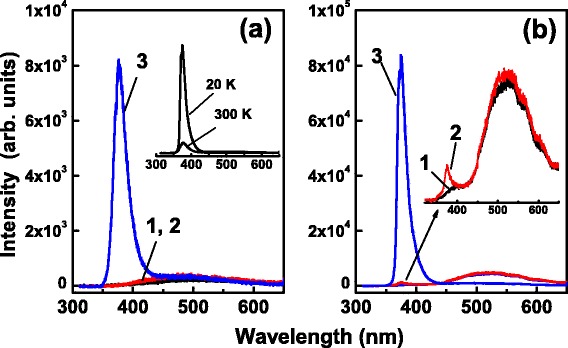
PL spectra of bare TiO_2_ NTs (*1*), 10-cycle ZnO/TiO_2_ NTs (*2*), and 25-cycle (*3*) ZnO/TiO_2_ NTs measured at 300 K (**a**) and 20 K (**b**). The inset in **a** compares the PL spectra of 25-cycle ZnO/TiO_2_ NTs measured at 300 and 20 K, and the inset in **b** shows magnified spectra 1 and 2 recorded at 20 K

Figure 6a shows the PL spectra of the samples measured at 300 K. For the 25-cycle ZnO/TiO_2_ NTs, an intense UV luminescence peaking at about 376 nm is observed, with very weak visible emission associated with defects such as oxygen vacancies [33, 34]. This UV luminescence is emitted from the ZnO coatings and corresponds to the room-temperature free exciton-related near-band-edge (NBE) emission of ZnO [30, 35, 36]. The intense UV ZnO NBE luminescence and the weak defect-related emission reveal the high quality of the ZnO coating and low concentration of defects. Aside from weak defect-related emission, in contrast, no obvious luminescence can be observed from the bare TiO_2_ NTs and the TiO_2_ NTs covered by a 10-cycle deposit of ZnO coating. The former is expected to have low probability of radiative recombination processes due to the indirect band energy structure of TiO_2_ [37, 38]. For the latter, the light excitation of the ultrathin ZnO coating might be ineffective, and hence, the generation of electrons and holes in the ultrathin ZnO coating is limited due to its ineffective absorption of exciting photons. Additionally, the suppression of electron-hole recombination due to the efficient spatial separation of electrons and holes in the heterostructured ZnO/TiO_2_ reduces the luminescence from the ZnO coating.

Generally, the PL from the sample becomes stronger at low temperatures. The inset in Fig. 6a compares the PL spectra of the 25-cycle ZnO/TiO_2_ NTs measured at 300 and 20 K, respectively. At 20 K, the intensity of the UV ZnO NBE luminescence is increased about one order of magnitude for the 25-cycle ZnO/TiO_2_ NTs as a result of the freezing of phonons and the quenching of nonradiative recombination processes at low temperatures [30, 35, 39]. The UV ZnO NBE luminescence at low temperature is still without the company of defect-related visible emission, but with a narrowed width as compared to that recorded at room temperature, as shown in Fig. 6b which illustrates the PL spectra recorded at 20 K. For the bare TiO_2_ NTs, only the defect-related visible emission is observed. In addition to the defect-related visible emission, the PL of the 10-cycle ZnO/TiO_2_ NTs includes a weak UV NBE luminescence from the ZnO coating with its intensity less than 2 % of that of the 25-cycle ZnO/TiO_2_ NTs. The comparison of the PL spectra of the ZnO-coated TiO_2_ NTs with that of the bare TiO_2_ NTs, measured at room temperature and low temperature, suggests that the visible emission is most probable associated with the defects in TiO_2_ NTs. Moreover, the much weak PL from the 10-cycle ZnO/TiO_2_ NTs as compared to that from the 25-cycle ZnO/TiO_2_ NTs reveals a lower rate of radiative recombination of photogenerated electrons and holes in the 10-cycle deposit of ZnO than in 25-cycle deposit of ZnO, implying that ZnO/TiO_2_ NTs with a thinner rather than a thicker ZnO coating have more efficient spatial separation of photogenerated electrons and holes and hence exhibit better photoelectrochemical activity as will be described below.

**Fig. 7:**
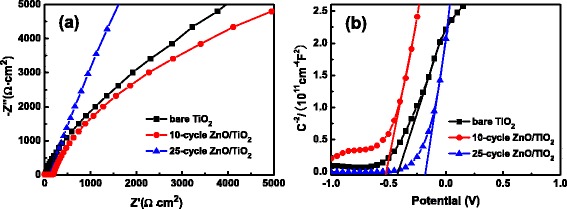
**a** Nyquist plots and **b** Mott–Schottky plots of bare TiO_2_ NTs, 10-cycle ZnO/TiO_2_ NTs, and 25-cycle ZnO/TiO_2_ NTs

From the typical electrochemical impedance spectroscopy (EIS) Nyquist plots of the bare TiO_2_ NTs and the heterogeneous structured ZnO-coated TiO_2_ NTs illustrated in Fig. 7a, we can determine the charge transfer resistance (*R*_ct_) of the samples when being used as electrodes. The charge transfer resistance of an electrode is one of the important parameters concerning the kinetics at the electrode. Compared to the bare TiO_2_ NTs, the TiO_2_ NTs covered by a 10-cycle deposit of ZnO present a decrease in *R*_ct_, as implied by the smaller value of the arc diameter in the Nyquist plot, indicating that the transfer of charges across the interface between the electrode and the solution becomes easier. However, the presence of the 25-cycle deposit of ZnO on the TiO_2_ NTs results in an increased *R*_ct_, even higher than that of the bare TiO_2_ NTs. The increase in *R*_ct_ is probably attributed to a longer transport path for charges because of the thicker ZnO coating on the TiO_2_ NTs and to a higher recombination rate of electrons and holes in the electrode, since the recombination rate in ZnO is higher than that in the TiO_2_ [40].

The flat-band potentials of the TiO_2_ NTs and ZnO/TiO_2_ NTs electrodes were determined by the Mott–Schottky analysis method [41, 42]. Figure 7b displays the Mott–Schottky plots of the bare TiO_2_ NTs and the ZnO-coated TiO_2_ NTs. The reversed sigmoidal plots with an overall shape are consistent with that typical for n-type semiconductors [42]. In addition, the reproducible flat-band potentials, i.e., the potentials corresponding to the situation in which there is no charge accumulation in the semiconductor so that the energy bands experience no bending can be obtained by intersecting the tangent of Mott–Schottky curves with the potential axis. The flat-band potential (*V*_fb_) for the 10-cycle ZnO-coated TiO_2_ NTs shifts negatively to −0.53 V from −0.42 V for the bare TiO_2_ NTs, revealing a smaller barrier for charge transfer in the 10-cycle ZnO/TiO_2_ NTs. The thin ZnO coating on the TiO_2_ surface forms an inherent barrier layer to block electron transfer from the conduction band or trap sites of TiO_2_ to electrolyte, retarding charge recombination in the TiO_2_/electrolyte interface [43]. The *V*_fb_ plays an important role in photoelectrochemical performance. A more negative *V*_fb_ suggests a higher photoelectrochemical activity. When used as the photoelectrode in a photovoltaic device, for example, a higher open-circuit voltage can be expected, since the open-circuit voltage (*V*_oc_) of a photovoltaic device is generally determined by the offset between the quasi Fermi level of electrodes and the redox level of electrolyte [44]. However, the 25-cycle ZnO-coated TiO_2_ NTs present a smaller negative value of *V*_fb_, even smaller than that of the bare TiO_2_ NTs. This can also be attributed to the higher recombination rate in thicker ZnO coating.

**Fig. 8:**
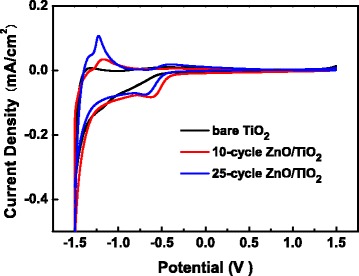
Cyclic voltammograms of bare TiO_2_ NTs, 10-cycle ZnO/TiO_2_ NTs, and 25-cycle ZnO/TiO_2_ NTs in a 0.5 M Na_2_SO_4_ solution at a scan rate of 50 mV/s

Figure 8 shows the cyclic voltammetry curves obtained for the bare TiO_2_ and the ZnO-coated TiO_2_ NTs electrodes in the 0.5 M Na_2_SO_4_ solution at a scan rate of 50 mV/s. All the cyclic voltammograms show hysteretic shape characteristics, which suggests that electron charging/discharging occurs in the electrode/electrolyte interface, forming Faradic currents in the electrolyte. Moreover, the ZnO coating on TiO_2_ induces a shift in the on-set potential of the cathodic current, consistent with the shift of flat-band potential, revealing that the ZnO coating plays a role as a blocking layer suppressing electron flow to the electrolyte. The shift of the on-set potential is also attributed to the changes of the interface states or traps on TiO_2_ surface covered by the ZnO coating.

**Fig. 9:**
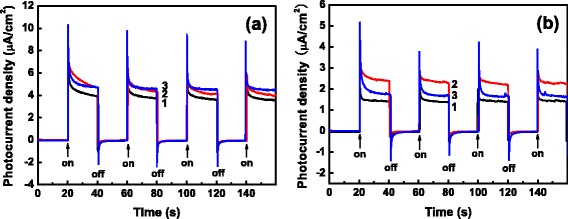
Transient PC densities of bare TiO_2_ NTs (*1*), 10-cycle ZnO/TiO_2_ NTs (*2*), and 25-cycle (*3*) ZnO/TiO_2_ NTs under illumination of light with wavelength cutoffs of 380 nm (**a**) and 420 nm (**b**)

Figure 9a illustrates the transient photocurrent responses of the ZnO-coated TiO_2_ NTs used as photoelectrodes under intermittent illumination by light with a wavelength cutoff of 380 nm and compares with that of the bare TiO_2_ NTs photoelectrode. Whether covered by a ZnO coating or not, the PC densities of the photoelectrode have a transient increase when the light on the electrode is turned on and decrease nearly to zero as soon as the incident light is turned off, demonstrating that the samples have a fast photoresponse speed and reasonably good photostability when used as photoelectrodes. Obviously, the coverage of the TiO_2_ NTs by ZnO coatings results in an increase in PC density and hence an enhancement in photoelectrochemical activity compared with the uncovered TiO_2_ NTs, and it seems that the increase of PC density and the enhancement of photoelectrochemical activity are nearly the same for the TiO_2_ NTs covered by a 10- or 25-cycle deposit of ZnO coating. The increased PC density and the enhanced photoelectrochemical activity of the ZnO-coated TiO_2_ NTs can be attributed to the nanotube-shaped TiO_2_ structure which has a large effective surface area in close proximity with the ZnO coating and consequently results in an increase of diffusive transport of photogenerated electrons and holes. In the heterogeneous structure composed of anatase TiO_2_ and wurtzite ZnO, in addition, an efficient spatial separation of photogenerated electrons and holes is expected, which also contributes to the increased PC density and the enhanced photoelectrochemical activity.

**Fig. 10:**
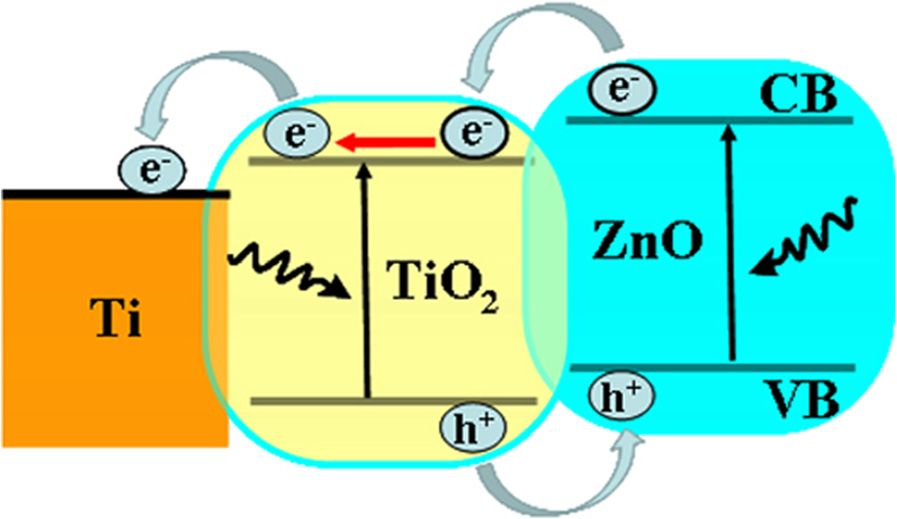
Band alignment of ZnO/TiO_2_ NTs grown on metallic Ti foil with schematic illustration for charge separation and transfer under illumination of light

The enhanced photoelectrochemical activity of the ZnO-coated TiO_2_ NTs can be better understood based on the staggered band alignment for the ZnO-coated TiO_2_ NTs on Ti foil as delineated in Fig. 10. Although the band gap energies of ZnO and TiO_2_ are similar (~3.37 vs ~3.22 eV), both the conduction band minimum (CBM) and valence band maximum (VBM) of ZnO lie a little above those of TiO_2_ [45], making a type-II heterojunction with a work function of about 5.2 eV for ZnO [46] and that of about 5.1 eV for TiO_2_ [47]. The contact between TiO_2_ and the underlying Ti foil is ohmic by nature, as the work function of Ti (4.33 eV) is less than that of TiO_2_. Therefore, when the nanoheterostructure is subjected to a positive bias, the electrons of the CB of ZnO can easily enter into the CB of TiO_2_ and then collected by the metallic Ti foil, whereas the holes of the VB of TiO_2_ can easily enter into the VB of ZnO. With excitation under light illumination, the photogenerated electrons in both TiO_2_ and ZnO reach at the interface between TiO_2_ and the Ti foil and are collected by the Ti foil, whereas the photogenerated holes reach at the ZnO surface where charge transfers occur between the electrolyte and the ZnO surface via electrochemical reactions.

The increased PC density and the enhanced photoelectrochemical activity can also be ascribed to a large number of electron-hole pairs formed under the illumination of the light around 380 nm the energy of whose photons is nearly resonant with the band gap of ZnO. As described above, however, the presence of the ZnO coatings on the TiO_2_ NTs results in a red shift of absorption edge and allows the ZnO-coated TiO_2_ NTs to absorb visible light. In order to investigate the influence of the ZnO coverage on the photoelectrochemical activity under visible illumination, PC measurements were also performed under intermittent visible illumination with a wavelength cutoff of 420 nm. Although the corresponding PC densities are generally lower than those obtained under the illumination with the wavelength cutoff of 380 nm, the ZnO-covered TiO_2_ NTs present enhanced photoelectrochemical activities compared with the bare TiO_2_ NTs. For the 10-cycle ZnO-covered TiO_2_ NTs, in particular, a significant enhancement of photoelectrochemical activity is observed. The PC density increases from ~1.4 μA/cm^2^ for the bare TiO_2_ NTs to ~2.2 μA/cm^2^ for the 10-cycle ZnO/TiO_2_ NTs, as shown in Fig. 9b. An increase of nearly 60 % in photoelectrochemical activity is therefore obtained for the TiO_2_ NTs covered by a 10-cycle deposit of ZnO, or by a 2.1-nm ZnO coating, compared with the bare TiO_2_ NTs. In contrast, the 25-cycle ZnO/TiO_2_ NTs just show a slight increase of PC density compared with the bare TiO_2_ NTs. One of the factors unfavorable for the enhancement in the photoelectrochemical activity of the 25-cycle ZnO/TiO_2_ NTs should be the thicker ZnO coating, which presents a longer pathway for the generated electrons to migrate to the TiO_2_ surface and the generated holes to the ZnO surface, and hence increases the recombination of electrons and holes, not conducive to the separation of electrons and holes. Apparently, the higher photoelectrochemical activity of the 10-cycle ZnO/TiO_2_ NTs is consistent with the suppressed recombination of electrons and holes in the heterogeneous nanostructures composed of TiO_2_ NTs and ultrathin ZnO coating. The above results suggest that from the view of enhancing photoelectrochemical activity, ZnO coatings of about 2 nm in thickness are optimal for heterostructured ZnO/TiO_2_ NTs [19, 40, 48, 49].

**Fig. 11:**
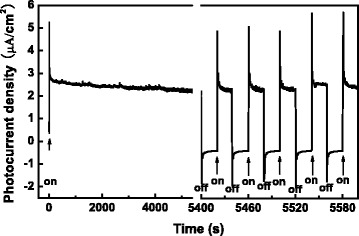
Transient PC density of 10-cycle ZnO/TiO_2_ NTs under visible illumination with wavelength cutoffs of 420 nm after a longtime illumination

The bare TiO_2_ NTs and ZnO-coated TiO_2_ NTs show good stability in the photoelectrochemical properties. As an example, Fig. 11 shows the PC density of the 10-cycle ZnO/TiO_2_ NTs for a longtime visible illumination. It is noted that no noticeable change is observed in the PC density after about one and a half hours of visible illumination of light with a wavelength cutoff of 420 nm and the 10-cycle ZnO/TiO_2_ NTs photoelectrode still presents prompt and excellent transient photocurrent responses under intermittent illumination. It has been reported that unlike ZnO films, ZnO/TiO_2_ NTs are stable in chemical properties and show excellent photostability [45].

## Conclusions

Vertically aligned ZnO-coated TiO_2_ NTs were fabricated by ALD deposition of ultrathin ZnO coatings on the walls of the TiO_2_ NTs formed by electrochemical anodization of Ti foils. The TiO_2_ NTs exhibit anatase structure while the ZnO coatings are structured with hexagonal wurtzite with precise thickness control at atomic scale for an investigation on the influence of ZnO coating and its thickness on the optical, electrochemical, and photoelectrochemical properties of the heterogeneous nanostructured ZnO/TiO_2_ NTs. The fabricated bare TiO_2_ NTs and the ZnO-coated TiO_2_ NTs present fast photoresponse and good photostability. Compared with bare TiO_2_ NTs, the presence of ZnO coatings significantly improves the electrochemical and photoelectrochemical activities due to the enhanced charge separation in the heterostructure composed of TiO_2_ NTs and ZnO coatings, especially the TiO_2_ NTs covered by a thinner ZnO coating of about 2 nm in thickness rather than a thicker ZnO coating. In comparison to the bare TiO_2_ NTs, an increase of nearly 60 % in photoelectrochemical activity is obtained for the TiO_2_ NTs covered by an about 2-nm-thick ZnO coating under visible illumination with a wavelength cutoff of 420 nm.
